# Analysis of bone erosions in rheumatoid arthritis using HR-pQCT: Development of a measurement algorithm and assessment of longitudinal changes

**DOI:** 10.1371/journal.pone.0265833

**Published:** 2022-04-26

**Authors:** Kazuteru Shiraishi, Ko Chiba, Kounosuke Watanabe, Nozomi Oki, Naoki Iwamoto, Shoken Amano, Akihiko Yonekura, Masato Tomita, Masataka Uetani, Atsushi Kawakami, Makoto Osaki

**Affiliations:** 1 Department of Orthopedic Surgery, Nagasaki University Graduate School of Biomedical Sciences, Nagasaki, Japan; 2 Department of Radiological Science, Nagasaki University Graduate School of Biomedical Sciences, Nagasaki, Japan; 3 Division of Advanced Preventive Medical Sciences, Department of Immunology and Rheumatology, Nagasaki University Graduate School of Biomedical Sciences, Nagasaki, Japan; Nippon Medical School, JAPAN

## Abstract

**Purpose:**

The purpose of this study was to establish an algorithm for measuring bone erosions at metacarpophalangeal (MCP) joints using high-resolution peripheral quantitative computed tomography (HR-pQCT), to investigate the precision of measurements, and to assess longitudinal changes in bone erosions among patients with rheumatoid arthritis (RA).

**Methods:**

The 2nd and 3rd MCP joints were scanned at a voxel size of 60.7 μm using second-generation HR-pQCT. Bone erosions on MCP joints were identified using a semi-automated algorithm we developed, and each erosion parameter was measured. Measurement reproducibility was evaluated in 19 healthy subjects using intraclass correlation coefficients (ICCs) and root mean square percent coefficient of variance (RMS%CV). Finally, longitudinal changes in bone erosions over a period of 12 months were assessed in 26 patients with RA based on the calculated least significant change (LSC).

**Results:**

Reproducibilities for measurement parameters regarding bone erosions with our algorithm were good (all ICCs ≥ 0.98; all RMS%CVs < 5%). No erosion parameters showed significant changes after 12 months of treatment in terms of median values in all erosions, while both progression and repair of erosions were observed individually (e.g., erosion volume: progression, 26% (+0.62 mm^3^); repair, 34% (-0.85 mm^3^); no change, 40%).

**Conclusions:**

The measurement algorithm developed for bone erosions at MCP joints showed good reproducibility. Both progression and repair of bone erosions were observed in patients with RA even after 12 months of appropriate treatment. Our algorithm may be useful to investigate the etiology of RA and assess drug efficacy.

## 1. Introduction

Rheumatoid arthritis (RA) is a systemic chronic inflammatory disease that causes cartilage and bone destruction in association with polyarthritis and leads to disability [1]. Bone erosion, as one of the characteristics of RA, is an important predictor for severity of disease and prognosis related to bone destruction [2, 3]. The identification of bone erosions in early RA and precise assessment on subsequent follow-ups are essential for diagnosis and treatment.

In recent clinical practice, computed radiography (CR) has been widely used to assess bone erosions [4]. Magnetic resonance imaging (MRI) and ultrasonography (US) are more effective for identifying bone erosions than CR [5]. However, CR only provides two-dimensional (2D) information, and MRI and US have limitations in providing detailed three-dimensional (3D) evaluations of bone.

High-resolution peripheral quantitative computed tomography (HR-pQCT) allows *in vivo* analysis of bone microarchitecture at peripheral sites with high spatial resolution [6, 7]. Currently, this modality is used for various studies in bone diseases, particularly in the field of osteoporosis [8, 9].

Research regarding RA using HR-pQCT has been increasing since around 2010 [10, 11]. In particular, HR-pQCT is advantageous for identifying small bone erosions compared to CR and MRI, because of the higher resolution [12]. Although bone erosions were clearly identified using HR-pQCT for not only RA patients, but also healthy subjects, indicating that bone erosions are not specific to RA [11, 13], bone erosions remain an important indicator in RA patients. Studies using HR-pQCT focusing on the analysis of longitudinal changes (progression and repair) in bone erosions are increasingly being conducted to verify the effects of medical treatments for RA [14–21]. On the other hand, several analytical methods have already been reported for identifying bone erosions and evaluating measurement parameters [22–25]. However, the precisions of these methods have not been fully established.

The main purposes of this study were to: 1) establish methods for analyzing bone erosions in metacarpophalangeal (MCP) joints using second-generation HR-pQCT; 2) verify the precision of parameters for measuring bone erosions using this algorithm; and 3) assess longitudinal changes in bone erosions over 12 months in RA patients applying this algorithm.

## 2. Methods

### 2.1. Subjects

Forty-eight healthy women with no history of finger arthritis were recruited to verify the precision of our algorithm in this study. Another 26 patients with RA fulfilling the 2010 American College of Rheumatology/European League Against Rheumatism (ACR/EULAR) classification criteria [26] participated in the longitudinal study and received treatments continuously with the same medications determined by a rheumatologist according to EULAR recommendations during the observation period.

Information on age, sex, height, weight, body mass index (BMI), disease duration, Disease Activity Score in 28 joints (DAS28), and the kind of medical treatment were recorded at baseline. At the same time, C-reactive protein (CRP), erythrocyte sedimentation rate (ESR), rheumatoid factor (RF), and anti-citrullinated protein antibodies (ACPA) were also measured. In addition, CRP, ESR, and DAS28 at follow-up were evaluated.

This study protocol was approved by Nagasaki University Hospital Clinical Research Ethics Committee (registration numbers: 16020828 and 18021923) and complied with the principles of the 1975 Declaration of Helsinki, as revised in 2000. Written informed consent was obtained from all participants prior to enrolment.

### 2.2. Scans

The 2nd and 3rd MCP joints of one hand were scanned using a second-generation HR-pQCT system (XtremeCT II; Scanco Medical, Bruttisellen, Switzerland) with reference to the scan protocol recommended by The Study group for xtrEme Computed Tomography in RA (SPECTRA) [27]. The dominant hand was scanned for healthy subjects and the affected hand was scanned for RA patients. The scan region was 20.4 mm in length, including the proximal phalanx and metacarpal head (MCH) of 2 fingers. Scan settings were as follows: voltage, 68 kVp; current, 1470 μA; power, 100 W; integration time, 43 ms; number of projections, 900; field of view, 140 mm; matrix, 2304×2304; voxel size, 60.7 μm; total number of slices, 336; and scan time, 4 min. The radiation dose was 10.8 mGy as the computed tomography dose index, and 22.0 mGy·cm as the dose-length product.

In healthy subjects, the same hand was scanned three times with repositioning on the same day, to verify the precision of our algorithm. In patients with RA, the affected hand was scanned at baseline and at the 12-month follow-up. Image quality was evaluated according to the motion artifact grading [28], and images with grade 4–5 were excluded from subsequent analysis.

### 2.3. Identification of bone erosions

In this study, erosions at the MCH and phalangeal base (PB) of the 2nd and 3rd MCP joints were analyzed using bone microstructure measurement software (TRI/3D-BON; Ratoc System Engineering, Tokyo, Japan).

**Fig 1:**
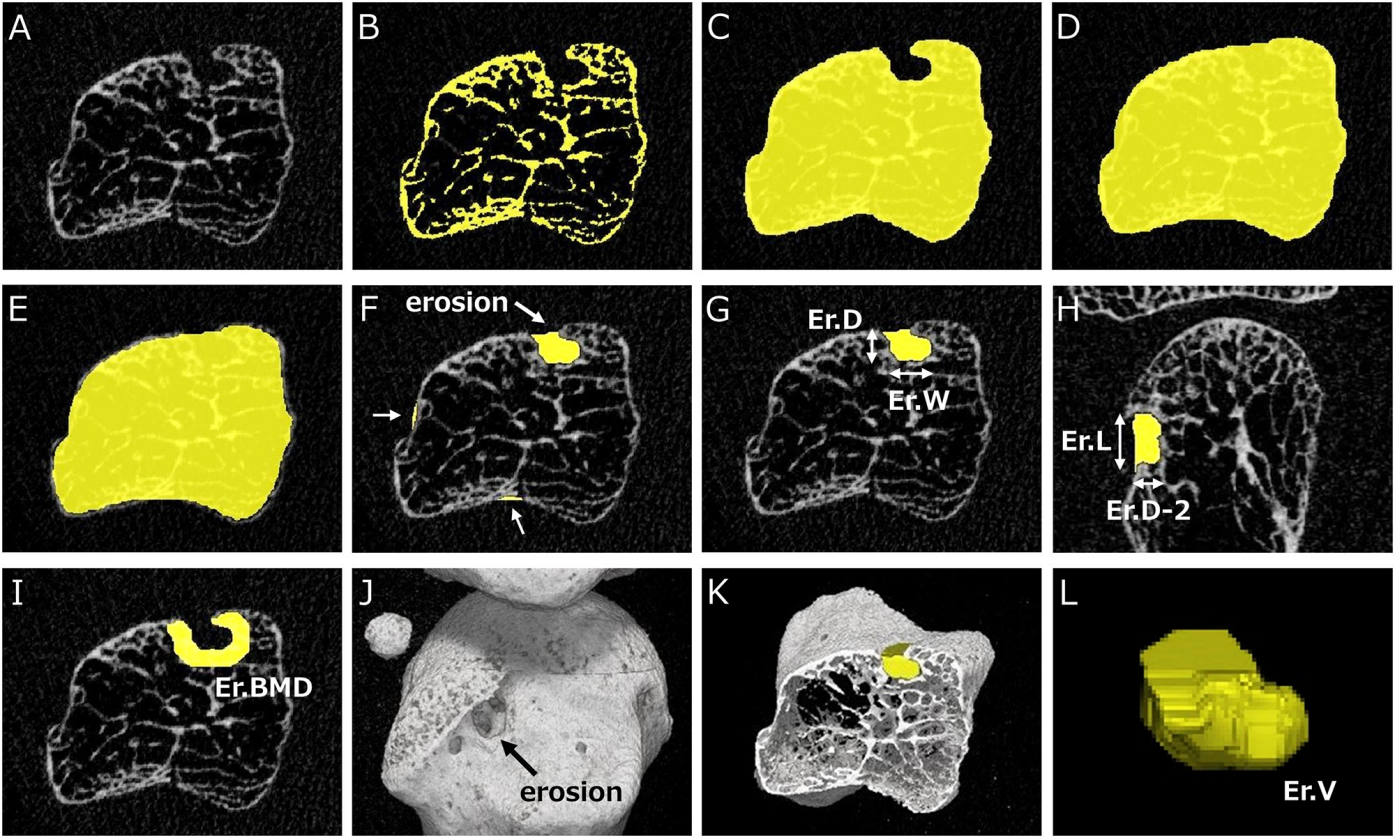
Algorithm for image analysis of erosion parameters for metacarpophalangeal (MCP) joints. A. Gray-scale image of the metacarpal head in the axial view. B. Binarization at a threshold value of 320 mg/cm^3^. C. Filling bone marrow spaces. D. Filling concavities on bone surface. E. Shrinking the contour from Fig 1D by 0.25 mm. F. Extraction of all concavities (white arrows) and detection of erosions based on the false-positive pattern in Fig 2 and the definition from SPECTRA. G. Manual measurement of erosion depth (Er.D) and width (Er.W) in the axial view. H. Manual measurement of erosion depth (Er.D-2) and length (Er.L) in the perpendicular (coronal) view. I. Measurement of peripheral BMD (Er.BMD) surrounding an erosion at a width of 1 mm. J. 3D image of MCH and erosion on the radial side (black arrow). K. Axial section of the 3D image including an erosion. L. Volume of interest (VOI) of an erosion and measurement of erosion volume (Er.V).

**Fig 2:**
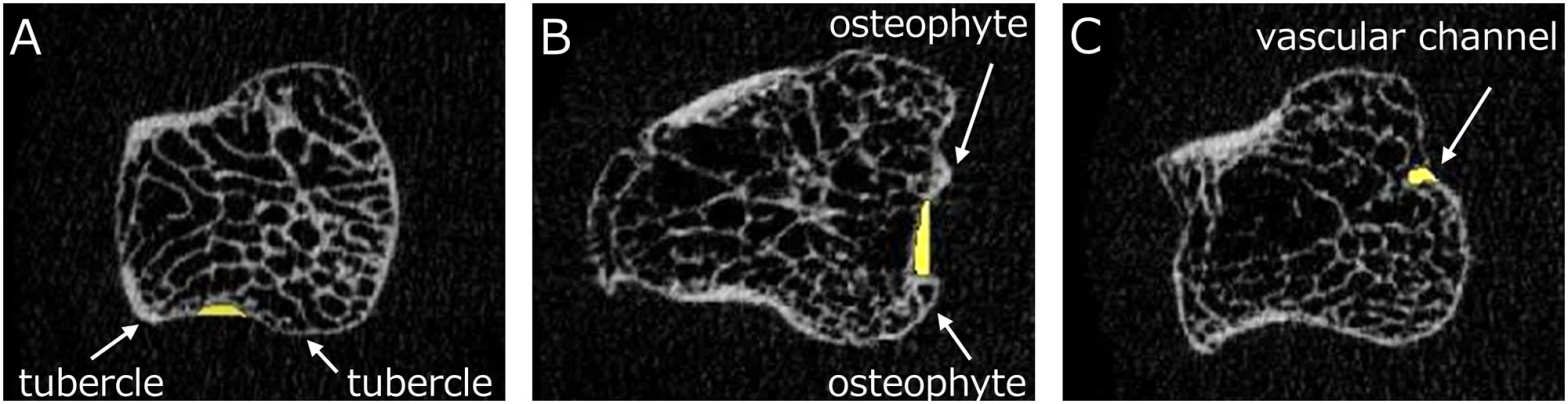
Typical false-positive patterns of erosions in our algorithm. A. Physiological concavity between tubercles (white arrows: tubercles). B. Pseudo-concavity formed by osteophytes (white arrows: osteophytes). C. Small cortical interruption or vascular channel (white arrow: vascular channel).

An overview of our algorithm for identifying and analyzing bone erosions is shown in Fig 1. First, an image of the MCH or PB to be analyzed was selected (Fig 1A) and a binarized image was created using a threshold value of 320 mg/cm^3^, which is a standard value for trabecular bone in second-generation HR-pQCT (Fig 1B). The bone mask image was then automatically created by filling the bone marrow space (Fig 1C). Subsequently, all concavities on the bone surface were automatically filled (Fig 1D), and the contour of this image was shrunk by 0.25 mm (Fig 1E). All concavities on the bone surface were then extracted by subtracting Fig 1C from Fig 1E (Fig 1F). As shown in Fig 2, the following concavities were excluded as false-positive patterns: physiological concavities (Fig 2A); pseudo-concavities formed by osteophytes (Fig 2B); and small cortical interruptions or vascular channels (Fig 2C). Finally, the remaining concavity was determined to represent a bone erosion after checking on a 2D axial view (Fig 1G), a coronal or sagittal view (Fig 1H), and a 3D view (Fig 1J and 1K) with reference to the definitions of erosion defined by SPECTRA [22].

### 2.4. Assessment of identified bone erosions

The volume of bone erosion was automatically calculated in voxel units based on the extracted volumes of interest (VOIs) (Fig 1L). Widths and depths of bone erosions were manually measured based on 2D images of the VOI. The depth and width of bone erosion in the axial view were defined as Er.D and Er.W, respectively (Fig 1G). In addition, depending on the distribution of erosions based on four quadrants, as published earlier [11], the depth and length of bone erosion in the perpendicular (coronal or sagittal) view were defined as Er.D-2 and Er.L, respectively (Fig 1H). (i.e., bone erosion on the radial or ulnar side was measured in the coronal view, and bone erosion on the dorsal or palmar side was measured in the sagittal view). In addition, peripheral bone mineral density surrounding bone erosion (Er.BMD) was automatically measured based on the VOI extracted with a width of 1 mm (Fig 1I) [24].

### 2.5. Additional procedures

**Fig 3:**
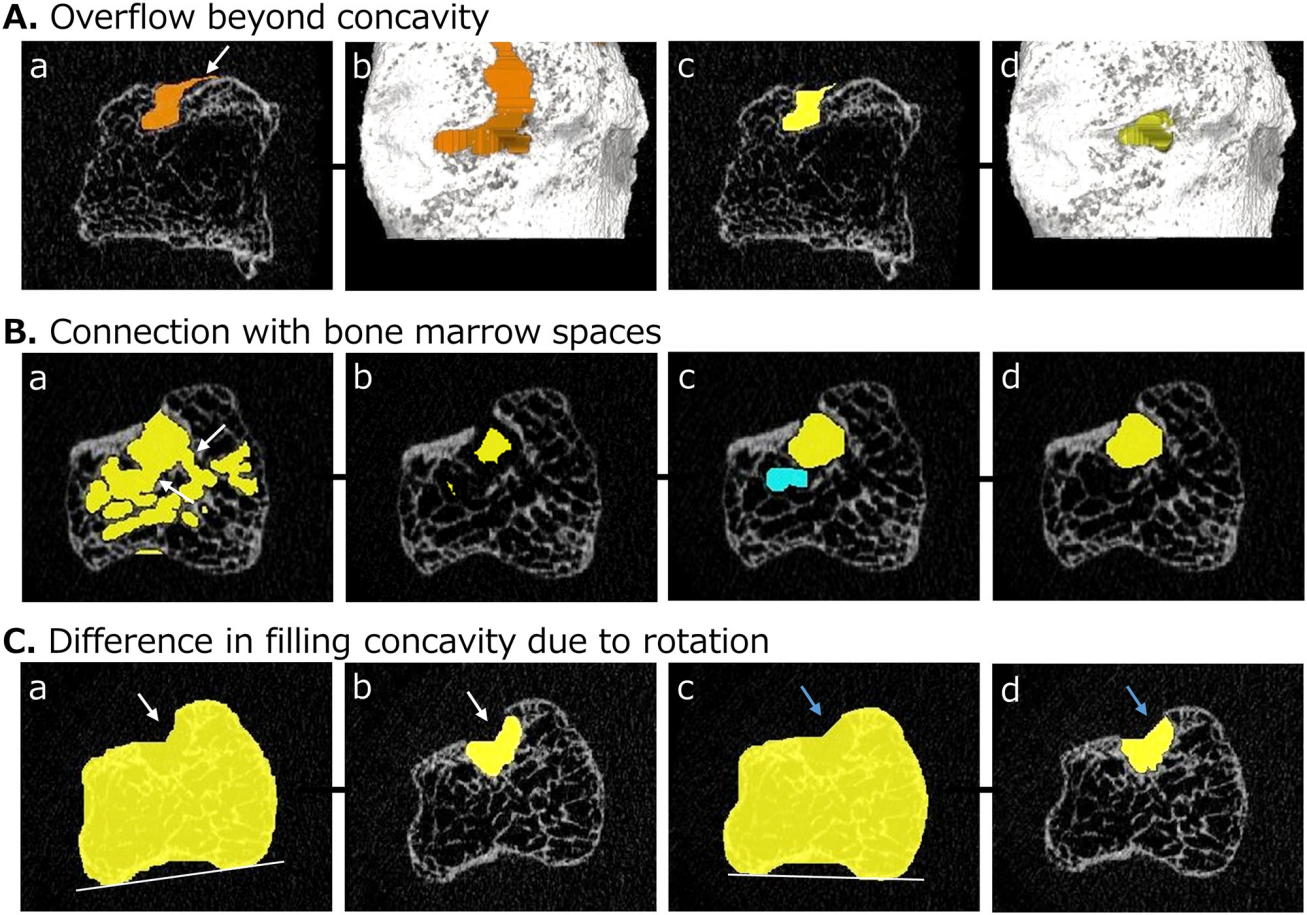
Additional procedures required for some erosions in our algorithm. A. This step may be necessary if the erosion VOI overflows beyond the concavity. a, b) These images represent overflow of the VOI (white arrow) on 2D (a) and 3D (b) images. c, d) The appropriate VOI is created by adding shrinking operations at the step in Fig 1E. B. These steps may be necessary if the erosion VOI connects with bone marrow spaces. a) The primary VOI is connected with the bone marrow spaces. b) The shrinking operation is executed iteratively until the continuity is completely interrupted. c) The dilation operation is executed the same number of times until a return to the original shape is achieved. d) Only the VOI of the erosion is extracted. C. Adjustment of rotation in the axial view may be necessary if the rotation is markedly different in longitudinal analysis. a, c) These images corresponding to the image in Fig 1D are created from the same image. A different rotation causes a slight difference in filling concavities. b, d) The two VOIs of erosions are slightly different due to differences in rotation.

Additional procedures are necessary for some erosions, as shown in Fig 3.

First, the step shown in Fig 3A may be necessary if overflow of the VOI beyond the concavity occurs (Fig 3Aa and 3Ab). In this case, the appropriate VOI was extracted by shrinking the contour of the image corresponding to Fig 1E by 0.50 mm (Fig 3Ac and 3Ad), which was applied because values above 0.50 mm made the VOI too small.

Second, the steps shown in Fig 3B may be necessary if the VOI connects with bone marrow spaces (Fig 3Ba). In this case, the VOI needs to be temporarily and repeatedly reduced in size until the continuities are completely interrupted (Fig 3Bb) [24]. Dilation operations are then executed until the VOI returns to its original shape (Fig 3Bc). Finally, the newly selected VOI is defined as the VOI of a bone erosion (Fig 3Bd). To ensure measurement reproducibility, the same number of size-reduction operations in these processes is used in longitudinal analyses.

Third, the adjustment of rotation in the axial view may be necessary if a certain degree of difference in rotation exists in the longitudinal analysis. As shown in Fig 3C, even using the same images, different rotation causes slight differences in filling concavities (Fig 3Ca and 3Cc) and the extracted VOIs (Fig 3Cb and 3Cd).

### 2.6. Statistical analysis

Intra-reader reliability for each bone erosion parameter using our algorithm was evaluated using the intraclass correlation coefficient (ICC) and root mean square percent coefficient of variance (RMS%CV) [29]. Least significant change (LSC) was calculated by multiplying the intra-reader precision error by 2.77, and both percent LSC (LSC of percent coefficient of variance: LSC%CV) and absolute LSC (LSC of standard deviation: LSCSD) were evaluated [30].

The Wilcoxson signed-rank test was used to assess longitudinal changes of bone erosions between baseline and 12 months follow-up in patients with RA. Er.BMD was evaluated only for bone erosions identified at baseline.

In addition, cumulative probability plots were created to analyze longitudinal changes of individual bone erosions in patients with RA. LSCSD calculated for each parameter was applied to these plots, and the percentages of the three categories of “progression”, “repair” and “no change” were calculated based on each LSCSD we regard as cut-off values. Similarly, percentages of these three categories (increasing, decreasing, and no change) for Er.BMD were calculated based on LSCSD for this parameter.

All statistical analyses were performed using JMP version 14 (SAS Institute Inc., Cary, NC, USA) and values of *p<*0.05 were considered significant.

## 3. Results

### 3.1. Measurement reproducibility of bone erosions

**Fig 4:**
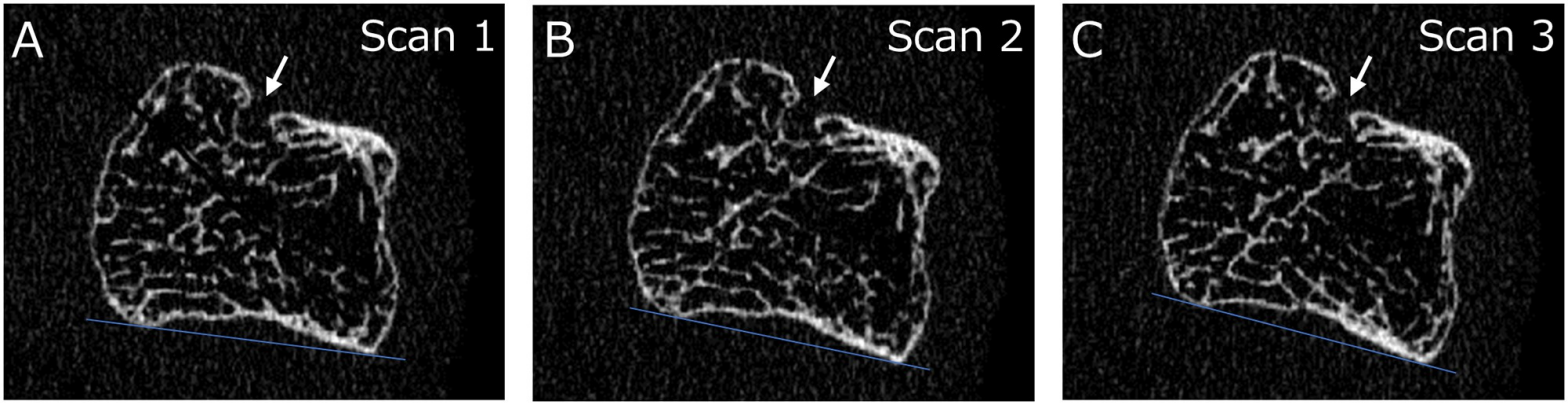
2D images of a metacarpal head in a healthy woman obtained from three scans with repositioning (white arrows: Erosion). A. 1st scan; B. 2nd scan; C. 3rd scan.

Twenty-five erosions were identified in 19 of 48 healthy subjects using our algorithm, and 15 erosions showing motion artifact of grade 1–3 in three scans in a row (Fig 4) were used to verify the reproducibility of measurement parameters. These results are shown in Table 1. ICCs of all parameters showed almost perfect reproducibility (0.98–1.00). RMS%CV of each parameter was less than 5% (1.91–4.65%), indicating good reproducibility. LSC%CV was 5.3% for Er.BMD, and 9.98–12.87% for all other parameters. LSCSD was 0.21 mm^3^ for Er.V and 14.7 mg/cm^3^ for Er.BMD, and 0.13–0.18 mm for width and depth of bone erosions.

**Table 1:** Measurement reproducibility of each erosion parameter.

	Mean (SD)	Median (IQR)	ICCs	RMS%CV (%)	LSC%CV (%)	LSCSD
Er.V (mm^3^)	2.09 (1.91)	1.69 (0.54–2.68)	1.00	3.97	10.99	0.21
Er.D (mm)	1.41 (0.67)	1.44 (0.79–1.74)	0.99	4.28	11.85	0.17
Er.D-2 (mm)	1.33 (0.52)	1.54 (0.83–1.74)	0.99	4.65	12.87	0.14
Er.W (mm)	1.71 (0.61)	1.58 (1.27–2.10)	0.98	3.66	10.15	0.18
Er.L (mm)	1.43 (0.41)	1.27 (1.11–1.68)	0.98	3.60	9.98	0.13
Er.BMD (mg/cm^3^)	308.1 (89.1)	277.4 (227.7–377.4)	1.00	1.91	5.30	14.7

Three scans and measurements were repeated for the reproducibility analysis. Values are calculated for precision error and displayed as mean and standard deviation (SD), median and interquartile range (IQR), intraclass correlation coefficient (ICC), root mean square percentage coefficient of variance (RMS%CV), least significant change with percentage (LSC%CV), and absolute least significant change (LSCSD).

Er.V, erosion volume; Er.D, erosion depth in the axial view; Er.D-2, erosion depth in the perpendicular (coronal or sagittal) view; Er.W, erosion width in the axial view; Er.L, erosion length in the coronal or sagittal view; Er.BMD, peripheral bone mineral density surrounding erosion.

### 3.2. Characteristics of patients with RA

Characteristics of patients with RA (20 women, 6 men) at baseline are shown in Table 2. Mean age was 66.5 ± 9.2 years, and disease duration was 108.9 ± 143.6 months. The numbers of patients divided into disease durations of <12 months, 12–120 months, and >120 months were 8, 10, and 8, respectively. Mean height was 1.56 ± 0.08 m, weight was 54.5 ± 10.9 kg, and BMI was 22.3 ± 3.9 kg/m^2^. RF-positive results were seen in 88% of patients, and ACPA-positive results in 50%. Mean parameters at baseline and follow-up were 0.69 and 0.11 mg/L for CRP, 33.9 and 15.9 mm/h for ESR, and 3.7 and 1.7 for DAS28-CRP, respectively. Methotrexate was being administered to 54% of patients, and biologics to 81%. In addition, 27% of patients were using low-dose glucocorticoids.

**Table 2:** Characteristics of RA patients (n = 26).

	Baseline	Follow-up (12months)
Age, years (SD)	66.5 (9.2)	-
Female, n (%)	20 (77)	-
Height, m (SD)	1.56 (0.08)	-
Weight, kg (SD)	54.5 (10.9)	-
BMI, kg/m^2^ (SD)	22.3 (3.9)	-
Disease duration, months (SD)	108.9 (143.6)	-
< 12 months, n (%)	8 (31)	-
12–120 months, n (%)	10 (38)	-
> 120 months, n (%)	8 (31)	-
CRP, mg/L (SD)	0.69 (0.89)	0.11 (0.15)
ESR, mm/h (SD)	33.9 (23.0)	15.6 (11.3) ^a^
RF-positive, n (%)	21 (88)^a^	-
ACPA-positive, n (%)	11 (50)^b^	-
DAS28-CRP (SD)	3.7 (1.2)	1.7 (0.8)
Use of MTX, n (%)	14 (54)	-
Use of biologics, n (%)	21 (81)	-
ETN, n (%)	1 (4)	-
ADA, n (%)	3 (12)	-
TCZ, n (%)	4 (15)	-
ABT, n (%)	12 (46)	-
CZP, n (%)	1 (4)	-
Use of low-dose glucocorticoids, n (%)	7 (27)	-

All data are shown as mean and standard deviation (SD), or absolute number and percentage.

RA, rheumatoid arthritis; BMI, body mass index; CRP, C-reactive protein; ESR, erythrocyte sedimentation rate; RF, rheumatoid factor; ACPA, anticitrullinated protein antibodies; DAS28, Disease Activity Score 28; MTX, methotrexate; ETN, etanercept; ADA, adalimumab; TCZ, tocilizumab; ABT, abatacept; CZP, certolizumab.

^a^ Values for two patients are missing

^b^ Values for four patients are missing

### 3.3. Longitudinal changes of bone erosions in patients with RA

Longitudinal changes of bone erosions between baseline and follow-up (12 months) in patients with RA are shown in Table 3. Thirty-four bone erosions (MCH: 23; PB: 11) were confirmed at baseline, and 37 (MCH: 26; PB: 11) were confirmed at follow-up, including 4 new erosions and complete repair of 1 erosion. For the total cohort, no significant changes between baseline and follow-up were seen for any parameter.

**Table 3:** Longitudinal changes in erosion parameters for patients with RA (n = 26).

		(1) Baseline	(2) Follow-up		
Total number of erosions		34	37		
		MCH: 23; PB: 11	MCH: 26; PB: 11		
			(new: 4; complete repair: 1)		
	Changes of each erosion			Difference (2)-(1)	
	n	Median (IQR)	Median (IQR)	Median (IQR)	*p*-value
Er.V (mm^3^)	Total	1.67 (0.63, 5.79)	2.15 (0.85, 6.23)	-0.03 (-0.51, 0.21)	0.61
	Progression: 10	0.67 (0.12, 1.97)	2.45 (1.13, 5.63)	0.62 (0.42, 0.95)	
	Repair: 13	5.74 (1.77, 10.4)	4.88 (0.86, 8.80)	-0.85 (-1.66, -0.57)	
Er.D (mm)	Total	1.06 (0.85, 1.85)	1.15 (0.79, 1.78)	-0.03 (-0.12, 0.06)	0.73
	Progression: 7	0.00 (0.00, 0.67)	1.28 (0.94, 1.75)	0.61 (0.28, 1.52)	
	Repair: 9	1.82 (1.34, 2.06)	1.46 (0.73, 1.70)	-0.36 (-0.36, -0.24)	
Er.D-2 (mm)	Total	1.12 (0.79, 1.93)	1.21 (0.76, 1.96)	0.00 (-0.23, 0.12)	0.86
	Progression: 8	0.15 (0.00, 0.83)	1.52 (0.91, 1.87)	0.43 (0.29, 1.35)	
	Repair: 12	1.28 (0.96, 2.11)	0.94 (0.73, 1.78)	-0.39 (-0.55, -0.29)	
Er.W (mm)	Total	1.79 (1.20, 3.04)	1.94 (1.34, 3.00)	0.03 (-0.18, 0.24)	0.47
	Progression: 11	1.28 (0.00, 2.12)	2.19 (1.67, 2.94)	0.43 (0.30, 1.24)	
	Repair: 6	2.19 (1.59, 2.96)	1.58 (1.11, 2.55)	-0.61 (-0.94, -0.46)	
Er.L (mm)	Total	1.58 (1.26, 2.19)	1.73 (1.33, 2.79)	0.06 (-0.17, 0.30)	0.52
	Progression: 14	1.24 (0.24, 2.00)	1.94 (1.52, 3.43)	0.39 (0.30, 1.05)	
	Repair: 10	2.55 (1.67, 4.84)	1.73 (1.27, 3.29)	-0.36 (-1.18, -0.24)	
Er.BMD (mg/cm^3^)	Total	329.8 (242.1, 387.6)	337.1 (285.9, 402.0)	10.3 (-9.7, 38.1)	0.39
	Increasing: 14	249.5 (230.8, 277.7)	315.4 (269.2, 362.5)	60.2 (29.2, 88.1)	
	Decreasing: 8	410.0 (367.3, 473.7)	378.6 (335.2, 417.5)	-36.5 (-51.0, -26.7)	

Values at baseline and follow-up, and differences between them are displayed as median and interquartile range (IQR).

The Wilcoxon signed-rank test was used for comparisons of each value between baseline and follow-up (significant *p* < 0.05).

Erosions with new occurrence and complete repair were excluded from statistics regarding Er.BMD.

MCH, metacarpal heads; PB, phalangeal bases; Er.V, erosion volume; Er.D, erosion depth in the axial view; Er.D-2, erosion depth in the perpendicular (coronal or sagittal) view; Er.W, erosion width in the axial view; Er.L, erosion length in the perpendicular view; Er.BMD, peripheral bone mineral density surrounding erosion.

**Fig 5:**
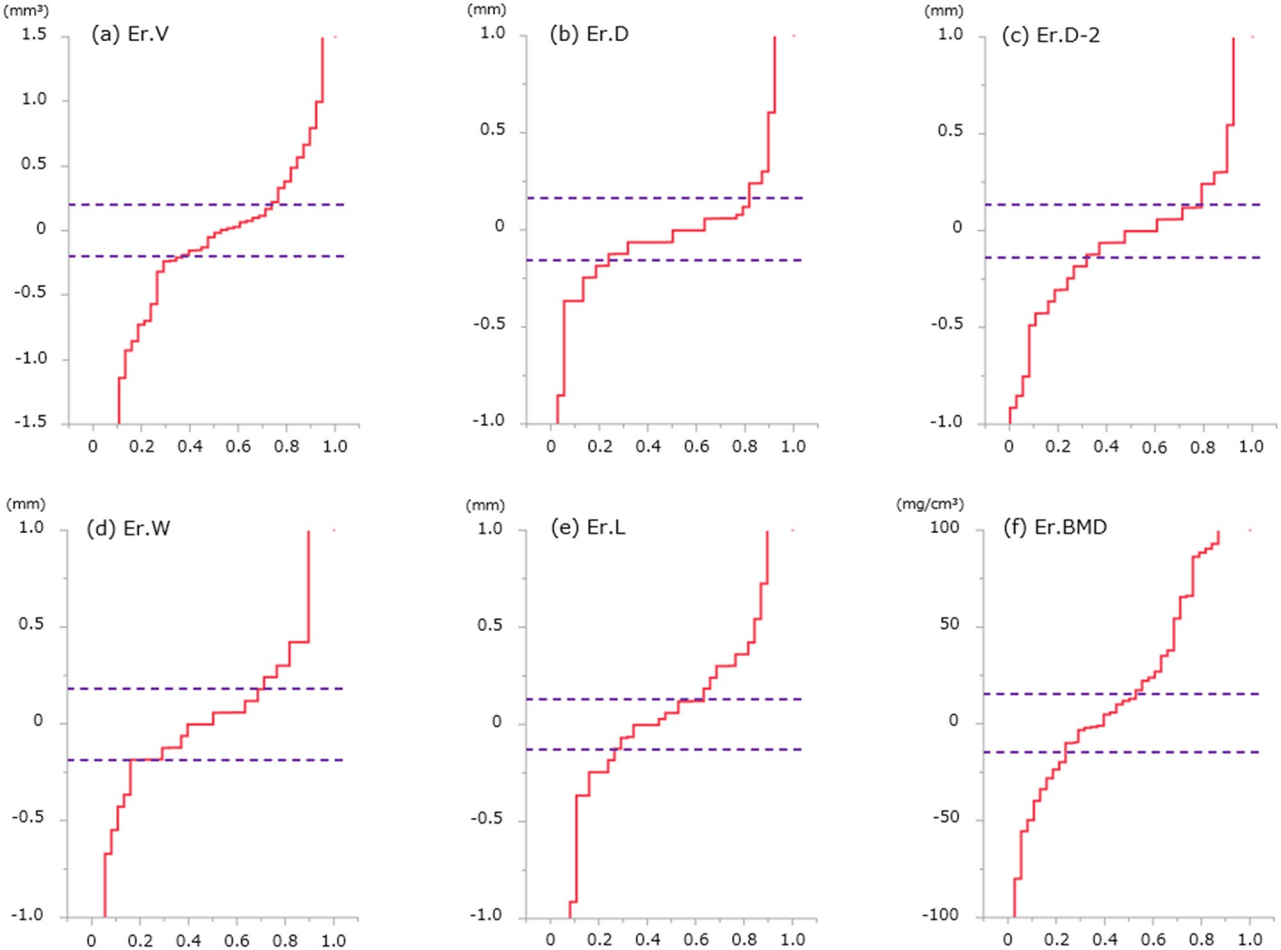
Cumulative probability plots regarding longitudinal changes in each erosion parameter for patients with RA. Each absolute least significant change (LSCSD) is shown as a broken line. Plots exceeding the LSC indicate significant changes on both positive and negative sides. (Er.V, erosion volume; Er.D, erosion depth in the axial view; Er.D-2, erosion depth in the perpendicular (coronal or sagittal) view; Er.W, erosion width in the axial view; Er.L, erosion length in the perpendicular view; Er.BMD, peripheral bone mineral density surrounding an erosion).

**Fig 6:**
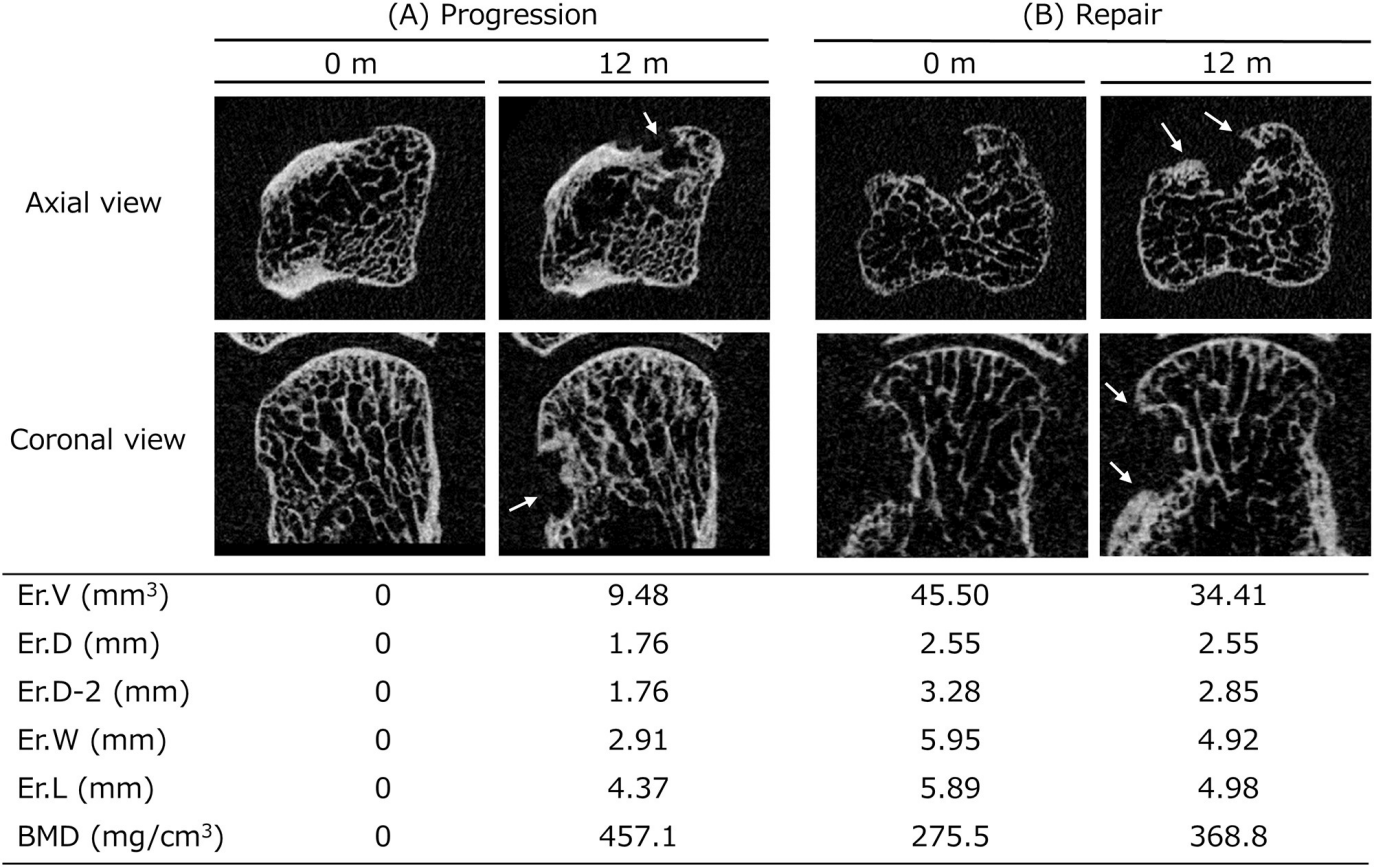
Two representative images of a longitudinal erosion change in patients with RA. Each 2D MCH image represents an image at baseline (0 months) and at follow-up (12 months) from both the axial view (upper) and the coronal view (lower). A. New erosion is evident in a 74-year-old patient (white arrows indicate new erosion). Clinical data at baseline were as follows: treatment with tocilizumab; disease duration, 60 months; rheumatoid factor (RF), positive; C-reactive protein (CRP), 0.89 mg/L; Disease Activity Score in 28 joints (DAS28)-CRP, 6.70. B. Partial repair of erosion is evident in a 49-year-old patient (white arrows indicate new bone formation). Clinical data at baseline were as follows: treatment with adalimumab; disease duration, 24 months; RF, negative; CRP, 1.42 mg/L; DAS28-CRP, 3.95.

Longitudinal changes of each bone erosion are shown in Table 3 and Fig 5. Cumulative probability plots showed progression in 26% (+0.62 mm^3^), repair in 34% (-0.85 mm^3^), and no change in 40% for Er.V, progression in 18% (+0.61 mm), repair in 24% (-0.36 mm), and no change in 58% for Er.D; progression in 21% (+0.43 mm), repair in 32% (-0.39 mm), and no change in 47% for Er.D-2; progression in 29% (+0.43 mm), repair in 16% (-0.61 mm), and no change in 55% for Er.W; progression in 37% (+0.39 mm), repair in 26% (-0.36 mm), and no change in 37% for Er.L; and increase in 42% (+60.2 mg/cm^3^), decrease in 25% (-36.5 mg/cm^3^), and no change in 33% for Er.BMD. Representative images for longitudinal changes in bone erosions are shown in Fig 6.

## 4. Discussion

In this study, MCP joints were scanned using second-generation HR-pQCT (voxel size, 61 μm), and a semi-automated analysis algorithm was established for bone erosions. In addition, the precision of each parameter regarding bone erosions was verified, revealing good reproducibility (all ICCs ≥0.98; all RMS%CVs <5%). Finally, longitudinal changes of bone erosions after 12 months in patients with RA were investigated. Each parameter showed no significant changes in the total cohort, while both progression and repair of erosions were observed on an individual despite appropriate treatment.

Because HR-pQCT enables detailed analysis of bone erosions, changes of bone erosion volume (progression and repair) are the most commonly used indicators for disease activity or treatment efficacy in RA. However, methods for analyzing bone erosions currently vary between different research groups.

One method was to estimate erosion volume from an ellipsoid formula based on manual measurements of depth and width for a visually identified erosion. This approach has been in wide use from the beginning of RA research using HR-pQCT [22, 23]. In addition, semi-automated algorithms to improve measurement precision have been reported, such as the 3D segmentation technique described by Töpfer et al. [24], or the cortical interruption detection described by Peters et al. [25]. In terms of intra-reader reliability for assessing bone erosion volume, RMS%CV was 5.66% for the algorithm described by Töpfer et al. [24], and ICC was 1.00 for the algorithm described by Peters et al., although RMS%CV was not mentioned [25].

In terms of the measurement reproducibility of bone erosions in the present study, RMS%CV was 3.97% and ICC was 1.00 in terms of the intra-reader reliability of our algorithm for assessing bone erosion volume. These results were comparable with those for the other two algorithms. One of the strengths for our algorithm is the measurement of depth and width based on the VOI. Although seemingly the same as the manual measurement mentioned above, the VOI clearly delineates sites of bone erosion in our algorithm. The RMS%CV of depth and width was thus less than 5%, indicating high precision.

Regarding longitudinal changes of bone erosions in this study, no parameter for bone erosions showed significant changes between baseline and follow-up for the total cohort, while both progression and repair of erosions were observed at the individual level. In particular, the results in this study showed progression in 26%, repair in 34%, and no change in 40% for erosion volume.

Existing studies have reported variable results regarding longitudinal changes in bone erosion volume on follow-up after approximately 1 year in patients with RA. Töpfer et al. reported that erosions increased in 15%, decreased in 20% and were stable in 65% after an average of 1.2 years [31]. Peters et al. reported that mean values of cortical interruption parameters were not significantly changed after 1 year, while the proportions of joints showing repair, progression, and no change in volume of interruptions were 7%, 6% and 87%, respectively [20]. Yue et al. reported that a reduction in erosion volume was apparent after 1 year, and the proportions of patients with erosions showing progression, partial repair and no change were 32%, 20% and 48%, respectively [32]. Such findings may have resulted from differences in study designs, such as background characteristics including duration of disease or pharmacotherapies, along with different methods of analysis. Nevertheless, a certain proportion of longitudinal changes in volume showing both progression and repair in each erosion was observed even with appropriate treatment, and our study supported the findings of those previous studies.

We expect that the algorithm described here will contribute to pathological analyses of RA or the effects of specific pharmacotherapies. Some studies have already verified the effects of specific pharmaceutical agents such as denosumab and tocilizumab [17, 21], and our algorithm may contribute to similar research in the future.

This study has several limitations. First, we verified the precision of bone erosion measurement in healthy subjects, not in RA patients, due to the need to scan several regions such as the fingers, wrist, radius, and tibia on the same day, in consideration of the problems of scan time and radiation dose. Bone erosions in healthy subjects were considered similar to those in patients with RA, as shown in Fig 4, and have been used as controls in previous studies [11, 20]. Second, we were unable to analyze huge bone erosions connected to bone marrow spaces. We were also unable to identify and analyze erosions that were too small. Small erosions not identified by our algorithm were considered as minute vascular channels or cortical interruptions, not bone erosions. Finally, images with severe motion artifacts may require careful analysis because of the potential for insufficient contouring of the periosteal line, particularly in longitudinal studies.

In conclusion, we established an analytical algorithm for bone erosions on MCP joints using second-generation HR-pQCT. Good reproducibility was obtained with our algorithm for measurement parameters regarding bone erosions. We observed both progression and repair of bone erosions at the individual level with longitudinal follow-up after 12 months in patients with RA, despite appropriate treatment. We believe that our algorithm allows precise assessment of bone erosions and may be useful for investigating the pathogenesis of RA and the effects of treatment.

## Abbreviations

2D: two-dimensional
3D: three-dimensional
ACPA: anti-citrullinated protein antibodies
BMI: body mass index
CR: computed radiography
CRP: C-reactive protein
DAS28: Disease Activity Score in 28 joints
Er.BMD: peripheral bone mineral density surrounding bone erosion
Er.D: depth of bone erosion in the axial view
Er.D-2: depth of bone erosion in the perpendicular (coronal or sagittal) view
Er.L: length of bone erosion in the perpendicular (coronal or sagittal) view
Er.W: width of bone erosion in the axial view
ESR: erythrocyte sedimentation rate
HR-pQCT: high-resolution peripheral quantitative computed tomography
ICCs: intraclass correlation coefficients
LSC%CV: least significant change of percent coefficient of variance
LSC: least significant change
LSCSD: least significant change of standard deviation
MCH: metacarpal head
MCP joint: metacarpophalangeal joint
MRI: magnetic resonance imaging
PB: phalangeal base
RA: rheumatoid arthritis
RF: rheumatoid factor
RMS%CV: root mean square percent coefficient of variance
SPECTRA: Study group for xtrEme Computed Tomography in RA
US: ultrasonography
VOI: volume of interest
